# “Hurt People Hurt Other People”: The Link Between Past Trauma and Sexual Offending

**DOI:** 10.5964/sotrap.7361

**Published:** 2022-03-31

**Authors:** Melissa D. Grady, Jill S. Levenson, Jessica Glover, Shelley Kavanagh, Katharine Carter

**Affiliations:** 1 National Catholic School of Social Service, Catholic University of America, Washington, DC, USA; 2 School of Social Work, Barry University, Miami Shores, FL, USA; 3 Private Practice, Fort Lauderdale, FL, USA; Simon Fraser University, Burnaby, BC, Canada

**Keywords:** trauma, sexual offending, trauma-informed care, qualitative research

## Abstract

**Background:**

A growing body of research documents the high rates of trauma among individuals who have sexually offended. Yet the relationship between prior victimization and subsequent sexual offending remained unclear. Objective: By including the voices and perspectives of individuals convicted of sexual offenses, we sought to strengthen professionals’ understanding of the connection between victimization and offending.

**Method:**

This qualitative study used an online survey to collect data from individuals convicted of sexual offenses (n *=* 195) with the aims of understanding their perceptions of the link between trauma and offending and what they would like sex-offense treatment providers to know about this connection.

**Results:**

Using grounded theory, five major themes emerged from the data analysis: Relationship between Trauma Offending (n = 91), Acknowledging the Connection (n = 57), Specific Effects of Trauma (n = 48), Individualized Treatment (n = 34), and Recognition of Humanity (n = 26).

**Conclusions:**

The participants perceived a strong connection between one’s own victimization and subsequent sexual offending. In addition, they offered specific recommendations for treatment providers, including individualizing treatment with an emphasis on humanity and compassion. Implications for trauma-informed practice and policy are discussed.

## Highlights

- People who commit sex crimes perceive that there is a connection between their past trauma and sex offending.
- They want practitioners to acknowledge the trauma connection and help them understand it.
- They believe that individualized treatment would be more effective than group interventions.
- They want to be treated humanely and with dignity.

## Abstract (plain language summary)

**Background:**

In recent years there has been increased attention to the prevalence of trauma among individuals who commit sex crimes. However, it is still unclear how past traumas contribute to subsequent offending and how the manifestations of trauma may put some individuals at risk for committing sexual crimes.

**Why was this study done?:**

This study was designed to hear from the individuals themselves, whose voices are often not included in research, about how to understand the complex relationship between trauma and sexual offending. With such information, practitioners could then use this information to improve their interventions.

**What did the researchers find?:**

We found that clients welcomed and appreciated the opportunity to offer their thoughts and experiences. They provided many examples of how they believe their past traumas contributed to subsequent sexual offending. They noted that trauma can connect to risk factors associated with trauma, including cognitive and relational patterns, a lack of coping skills, confusion about sexual boundaries, and repetition of adverse childhood experiences. They also discussed their wish to increase their understanding of these experiences as a way to ensure that they do not repeat their behaviors again in the future. Furthermore, they expressed a strong desire to have therapists both acknowledge and address this connection. In addition, they advocated for more individualized treatment that is delivered with respect and dignity, which they believe would increase the overall effectiveness of interventions for preventing reoffense.

**What do these findings mean?:**

The findings indicate that practitioners working with this population may need to increase their knowledge in several areas. The first is in trauma-informed care to ensure that they provide a safe space for their clients to discuss and process their trauma and avoid retraumatization within the helping relationship, which some stated they experienced in the treatment in which they participated. Second, knowledge about the impacts of trauma should inform case conceptualization of relational patterns or risk factors that may have contributed to their offending and/or risk for future offending. Finally, given the high rates of trauma among this population, practitioners may want to consider learning more about trauma-specific interventions that target reduction of PTSD symptoms and maladaptive schemas, and improvement in coping skills.

Researchers, clinicians, and policy makers have long sought to understand the root causes of sexual violence. Recently, trauma has garnered significant attention as a potential contributor to the development of sexual offending (Creeden, 2009; Grady et al., 2017; Levenson, 2020) due to the well-documented high rates of adverse childhood experiences (ACEs) among individuals who have committed sexual crimes (Abbiati et al., 2014; Drury et al., 2017; Levenson et al., 2015; Levenson et al., 2016; Levenson, Baglivio, et al., 2017; Reavis et al., 2013). Research demonstrates the direct and indirect relationships between various ACEs and subsequent criminal behavior (Tripodi & Pettus-Davis, 2013; Wolff & Shi, 2012), including sexual offending (Grady, Yoder, et al., 2021; Marotta, 2021; Yoder et al., 2020; Yoder et al., 2019). While there is a growing body of literature, more research is needed to understand more clearly the role of trauma in the development of sexually violent behaviors in order to inform prevention and intervention efforts.

Developmental trauma (van der Kolk, 2005) is also known as complex trauma (Courtois, 2004; Ford et al., 2010) or relational trauma (Schore, 2003), and results from repeated and prolonged exposure to child abuse, neglect, and family dysfunction. Trauma can impact development in many ways. On a physical level, chronic toxic stress in childhood stimulates production of hormones that create a hyper-aroused state in which the brain scans the environment for danger; the body is prepared to respond to threats with a fight, flight, or freeze survival mode (van der Kolk, 2006). As a result, cognitive processing might be impaired, causing deficits in executive functions such as impulse control and strategic decision-making (Courtois & Ford, 2009; Ford et al., 2010; Schore, 2003). Interpersonally, children who grow up with insecure attachments might have poor models for healthy intimacy and effective relationship skills (Grady et al., 2017). Insecure attachments are also associated with emotional dysregulation, which can contribute to sexualized coping and congruence with minors (Stinson et al., 2008). As such, the neuro-cognitive and psychosocial consequences of early trauma are thought to contribute to the potential for sexual offending, although the exact mechanisms remain unclear.

One way clinicians and researchers can garner additional clarity about this connection is to learn from the perspectives of individuals who have committed sexual offenses. Furthermore, learning from the shared perceptions of people who committed sexual crimes is vital to understanding how they view and prioritize their treatment needs. By developing a more complete picture of the underlying and multi-faceted contributors to sexual offending, practitioners can enhance and improve prevention and intervention programs. The aim of this study was to explore how people who sexually offended perceive the connection between childhood experiences of trauma and subsequent sexually abusive behavior, and what they believe is important in the treatment process.

## Connections Between Childhood Adversity and Sexual Violence

Trauma is described as an event that feels threatening to a person’s physical safety or psychological well-being; the circumstances are usually out of a person’s control and can overwhelm their normal capacity to cope with stress (Substance Abuse and Mental Health Services Administration [SAMHSA], 2014a). Childhood adversity is not typically an isolated event, and it can therefore create a web of experiences by which a child organizes an understanding of self, others, and the world (Bloom, 2013). ACEs include child neglect, physical and sexual abuse, and family dysfunction such as domestic violence, addiction, absent parents, mental illness, and criminality (Felitti et al., 1998). Exposure to ACEs can stimulate distorted cognitive schemas, poor self-regulation capacities, and unhealthy attachment styles, all of which can contribute to risk for engaging in crime.

Compared to the general population, individuals who committed sex crimes reported higher rates of child sexual abuse, physical abuse, verbal abuse, and emotional neglect, and they were more likely to be raised by single parents in turbulent households (Drury et al., 2017; Levenson et al., 2016). Compared to other adolescents in the juvenile justice system, youth who sexually offended had significantly higher prevalence rates of physical abuse, sexual abuse, physical neglect, and household dysfunction (Levenson, Baglivio, et al., 2017). Moreover, researchers have found correlations between the number of adverse events experienced in childhood and the severity and frequency of violence and criminal conduct (Levenson & Grady, 2016; Levenson & Socia, 2016; Marotta, 2021). People with a criminal conviction for sex crimes tend to have higher rates of complex trauma, defined as the accumulation of multiple traumas that are often chronic and unrelenting (Courtois, 2004; Drury et al., 2017; Levenson et al., 2016; Levenson & Socia, 2016; Stensrud et al., 2019).

Traumatic events are experienced uniquely by each person, and their impacts will vary depending on the meaning attached to the experience and one’s resilient characteristics (SAMHSA, 2014a). Childhood trauma does not directly cause offending, but a higher level of early adversity is associated with early onset of sexual activity, and higher rates of sexually transmitted diseases, sexual delinquency, promiscuity, and sex work (Dietz et al., 1999; Hillis et al., 2000; Levenson, Willis, et al., 2017; Naramore et al., 2017). Thus, mistreated children are vulnerable to increased risk for sexually risky behavior and violating the boundaries of others.

### Risk Assessment and Clinical Conceptualization

In addition to actuarial risk assessments used to establish a baseline of risk, a focus on clinical case conceptualization (Craig & Rettenberger, 2018; Gannon & Ward, 2014; Levenson, Willis, et al., 2017) can help us theorize how childhood relational traumas might manifest later in aggressive, antisocial, manipulative, or paraphilic sexual behavior. Such case conceptualizations help therapists to better understand the origins of criminogenic needs and adjust service delivery to address dynamic risk factors in adherence to the responsivity principle (Jung, 2017). By understanding the life experiences that shape relational styles and disinhibition, we can replace a pathology-driven assessment of what is “wrong” with someone (SAMHSA, 2014a) with a more trauma-informed approach. Strengths-based and trauma-informed Risk-Needs-Responsivity (RNR) models help clients build coping and relationship skills that decrease risk and improve interpersonal functioning (Andrews & Bonta, 2010b; Bath & Seita, 2018; Willis et al., 2013; Yates et al., 2010).

Dysregulation resulting from early adversity can manifest in dynamic risk factors for sexual offending (Grady et al., 2017; Grady, Yoder, et al., 2021), which include antisocial attitudes, behaviours, or peers, sexual entitlement or preoccupation, intimacy deficits, impulsivity, poor decision-making, substance abuse, lack of involvement in pro-social activities, and negative or hostile moods (Hanson & Harris, 1998; Ward & Fortune, 2016). Dynamic risk factors and criminogenic needs reflect problems and characteristics that increase an individual’s chance of engaging in future crime, and they can be altered through interventions (Andrews & Bonta, 2010a). Therefore, the connection between early adversity and dynamic risk are relevant in our treatment programs (Levenson, Willis, & Prescott, 2017).

### Challenges in Qualitative Research With Individuals Who Commit Crimes

Historically, the voices of people who commit crimes have been relatively absent in the scholarly literature. A recent systematic literature review noted that only 11.3% of the 8,522 articles published between 2010 and 2019 in the top 17 criminal justice/criminology journals were qualitative in nature, and only 18 articles focused on individuals who had committed sexual crimes (Copes et al., 2020). This low number may be due to the numerous challenges to this type of research, which include accessing and recruiting participants, issues of confidentiality and anonymity, and providing informed consent according to U.S. federal guidelines for ethical research with prisoners who are defined as a vulnerable group (Blagden & Pemberton, 2010). The potential biases of scholars and society may also limit the inclusion of consumer voices in criminal justice research. “Rarely do we seriously consider turning our scholarly lens on [understanding] the powerful oppressors or those who perpetrate symbolic and meaningfully real acts of violence” (Waldram, 2007, p. 967). Given the negative public perceptions regarding individuals who commit sexual crimes (A. J. Harris & Socia, 2016), it is possible that scholars may worry about the reputational consequences of giving voice to the humanity of those most feared and reviled. Waldram (2007) eloquently articulated these potential concerns:

How do we portray those who are violent, who cause harm to others? Do we condone, even encourage, evil when we talk about it? When we learn that, contrary to the beliefs of many, evildoers are not entirely evil, how do we communicate that in a manner that restores their life-representations to the appropriate level of complexity? (p. 967)

The importance of exploring the relationship between life experiences of secure forensic mental health care services users and their subsequent dangerous behavior is beginning to emerge in the literature (Cartwright et al., 2022). Helping clients make sense of these connections can further clinical case conceptualization models with an aim to improve service implementation (Cartwright et al., 2022). Forensic services interviewees described insightful themes that attempted to explain the nexus between past adversities and criminal conduct. They described chaotic lives of abuse, rejection, inferiority, lack of trust, and maladaptive efforts to seek acceptance, often reinforced through poor modeling by caregivers. Coping skills designed to manage painful emotions intersected with dysregulated behaviors that served to externalize their distress. They took responsibility for their adult actions that resulted in forensic care, but considered the distorted schemas that emerged from early adversity and contributed to harmful behaviors. Finally, some offered a poignant and somber reminder that children’s pain often goes unseen and unheard, leaving them unprotected; many illustrated how finally having support was instrumental in their healing (Cartwright et al., 2022).

When individuals who commit sexual crimes are interviewed in qualitative research, however, the focus has often been on exploring their experiences of treatment (Levenson et al., 2009), rather than investigating their insights or perspectives regarding their own criminal actions (Cooper & Holgersen, 2016; see Drapeau et al., 2005; Grady & Brodersen, 2008). However, two studies have explored the connection between these individuals’ past and their sexual offending, and their voices informed our research.

In the first study, 63 adult males convicted of child sexual abuse were interviewed to better understand the connections between their life experiences and engagement in crime (J. Sullivan & Sheehan, 2016). Based on the interviews, the authors identified three main themes that they believed developed through these men’s life experiences: *maladaptive perceptions*, such as views about the self or children; *activated sexual arousal*, which included inappropriate arousal or associations between negative stimuli and pain; and *developmental difficulties*, such as feeling socially awkward or being able to maintain healthy adult peer or romantic relationships. What is important to note, however, is that it was the researchers who developed the themes. The men were not asked to make the connections. Instead, it was the researchers who built the theories to explain the relationship between client histories and subsequent offenses.

A more recent study sought to explore the “sexually abused-sexual abuser” cycle using interviews of 41 men who had sexually abused children (Lambie & Reil, 2021, p. 1). Among the participants, about half did not believe there was a connection between their own histories and subsequent offending behaviors. They attributed the cause(s) of their offending to external sources, such as revenge and anger towards others. For those who did see a connection between their own “life-changing” (Lambie & Reil, 2021, p. 6) abuse and subsequent offending, they identified several ways in which their abuse contributed to their offending. These factors included a preoccupation with sex, being sexually confused, and having simultaneous feelings of disgust and pleasure associated with sex. They developed distorted thinking patterns about child sexual abuse, including the range of abusive behaviors used by those who offend, and empathy for those who have experienced sexual abuse. These individuals noted that the abuse they experienced also contributed to becoming oversexualized early in their development, which led them to later normalize sexual contact between adults and children. The authors concluded that sexual abuse of children was motivated by sexualization, externalization, and normalization.

While these two studies offer some speculative explanations, more research is needed to “explore offenders' stories of their formative life experiences, as well as the ways in which these events were assimilated, if one wishes to identify what may have motivated them” (Sullivan & Sheehan, 2016, p. 85). Such research is crucial in understanding the link between childhood adversity and subsequent sexual offending and to identify what may be helpful in the treatment process. “If we do not make an attempt to understand those who violate our social norms by perpetrating violence…then we are most certainly likely to fall victim again. Sometimes it is in our best interests to listen, however difficult that may be” (Waldram, 2007, p. 969).

## Current Study

This study sought out the voices of those who have been convicted of sexual crimes to learn what they perceive to be the connections between childhood adversity and subsequent sexual offending. We also asked what they believe is important for treatment providers to know about this connection when providing sex-offending specific treatment programming (SOTX). As such, the aim of the study was to inform our knowledge in these areas and improve service delivery.

## Method

This study utilized an online survey to gather qualitative and quantitative information regarding how individuals/clients who have been convicted of sexual crimes perceive the role of past trauma in their later offending. It was part of a larger project that included two parallel on-line surveys, one for clinicians and one for individuals who have sexually offended, that were deployed in mid-2019. In this paper, only the responses of the individuals convicted of sexual offenses are presented. This project was approved by two different Institutional Review Boards at the authors’ universities.

### Data Collection and Sampling Strategy

The sample consisted of individuals convicted of sexual offenses who had or were currently participating in a treatment program for sexual offending (we will sometimes refer to them in this paper as “clients”). The demographic information for the sample of SOTX clients (*n =* 195) is displayed in Table 1.

**Table 1:** Demographics of Clients

Client	Client (*M* or %)
**Age**	Valid *n* = 141; Range 20-81; *M* = 48.84 (*SD* = 13.72)
**Race/Ethnic**	Valid *n* = 144
White	88.2%
Black	2.8%
Hispanic	2.8%
Other	6.3%
Marital Status	Valid *n* = 143
Single, never married	30.1%
Married	35.7%
Separated	2.1%
Divorced	15.4%
Living w/Partner	7%
Long-term Relationship	5.6%
Widowed	3.5%
Gender	Valid *n* = 146
Male	95.2%
Female	4.8%
Education	Valid *n* = 145
Some HS	2.8%
HS grad	16.6%
Some college	34.5%
Bachelor’s degree	24.8%
Graduate degree	21.4%
**Employed**	Valid *n* = 144; 66.7%
**Parent**	Valid *n* = 145; 54.5%
**Living w/ Children**	Valid *n* = 107; 27.1%

Two different recruitment methods were used to reach potential SOTX participants using purposeful and snowball sampling strategies. The first was through the authors’ use of their professional contacts and memberships. Using this method, the two lead authors emailed different professional groups and individuals the links to the surveys; we asked clinicians to pass on the link to any clients they thought might be interested in participating. A second distribution method was directed at clients only. Two different organizations, the Florida Action Committee and the National Association for Rational Sexual Offense Laws, both agreed to post the survey invitation to their members. Although we did not make any attempts to only recruit from the United States, all participants came from this one country. All forms of the email invitations also included a pdf version of the survey that could be printed and completed by hand for people without internet access. In total, 19 clients opted to complete the paper version of the survey and their responses were entered into the database upon receipt. Participants were provided with information explaining the risks and benefits of participating in the study, and participants were asked to click or check (for paper version) a box indicating that they had read the information about the study, that they were an adult (18 or older), and that they consented to participate.

### Measures

This study included a limited number of quantitative questions. The first asked participants to answer the following question using a Likert scale ranging from 1-5: How important do you believe it is for therapists to include a focus on past traumas when addressing offending behaviors? In addition to this question, participants were asked to provide demographic information, along with a few questions related to three other areas: trauma histories; personal histories of substance use, mental health, health, arrests, and interpersonal violence; and information about their previous crimes, both sexual and non-sexual. The majority of the data presented in this paper stemmed from two qualitative questions, which were used to address the aims of this study: In general, how would you describe the relationship or connection between someone’s experiences with trauma and later sexual offending? and What do you think is important for therapists and other professionals who work with people who have sexually offended to know about the connection between trauma and sexual offending?

### Data Analyses

Univariate descriptive statistics were calculated for the quantitative question and demographics. The qualitative data were analyzed using the constant comparative method for a content analysis (Corbin & Strauss, 2015). Derived from grounded theory (Corbin & Strauss, 2015; Padgett, 2008), this method is used to develop concepts from the data by coding and analyzing at the same time while simultaneously developing theories (Glaser, 1965; Kolb, 2012; Olson et al., 2016).

We used Olson and colleagues’ 10-step process, specifically designed for multiple raters. Each researcher 1. performed open-coding of data; 2. collaborated to unify codes; 3. re-coded data using unified codes; 4. calculated inter-coder reliability (ICR); 5. collaborated to discuss each code and identified areas lacking agreement; 6. repeated the above process for each segment of the data, producing a unified codebook applicable to all data subsets; 7. re-coded all data, producing themes; 8. selected themes for further analysis; 9. conducted co-occurrence analysis; and 10. constructed an exploratory model – the findings of the study.

The research team consisted of the two lead authors and three graduate students who were trained in the method above and supervised by the lead author. In analyzing these data, two coders worked together using the steps above to achieve ICR of at least 90% for the two questions included in the study. Prior to beginning Olson and colleague’s (2016) process, each coder reviewed the data and developed an initial set of codes along with a corresponding conceptual definition. The research team then further refined these codes and definitions, yielding 32 codes that were used for the first round of coding. After steps 1 and 2 of Olson and colleague’s process, the ICR averaged 48%. Through conference the coders unified the codes, recoded, and recalculated the ICR two more times until they reached an ICR rate of 90% (89.99% on the first question and 91.94% for the second) using a final list of 20 codes. After the final round of coding, the research team members sorted the 20 codes into the final five major themes presented in the Results section. The total number of times each theme was mentioned is presented along with a description and illustrative quotes.

## Results

### Quantitative Results

**Figure 1:**
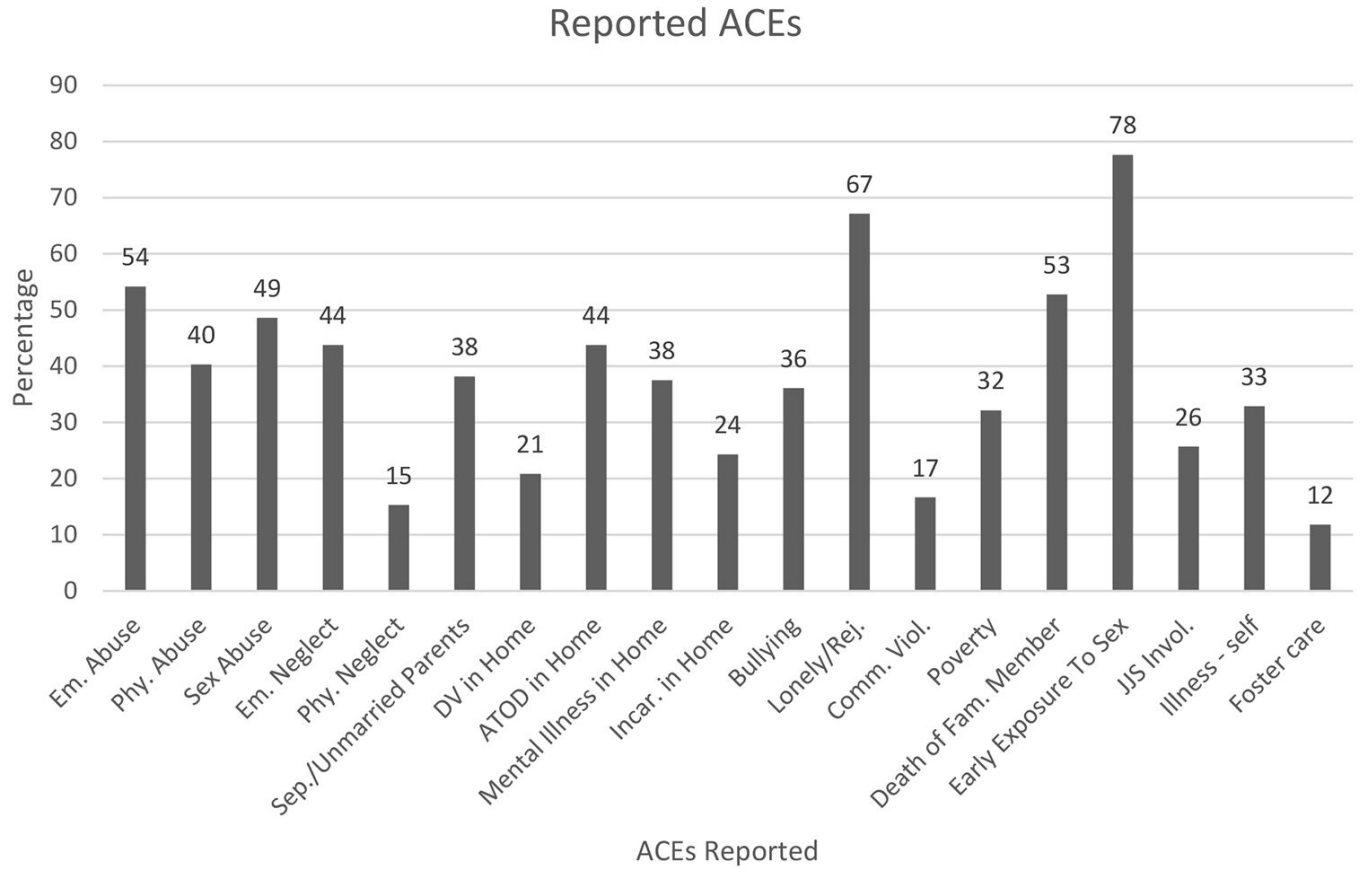
Percentage of Participants Reporting Each ACE *Note.* Table Key: em = emotional; phy. = physical; sep = separated; DV = domestic violence; ATOD = alcohol and other drugs; incar. = incarceration; rej. = rejected; comm. = community; fam = family; JJS = juvenile justice system.

The clients were asked about their experiences with nineteen different types of trauma before the age of 18 using a modified ACE scale. These dichotomous (yes/no) questions included various types of direct abuse (physical, sexual, neglect), household dysfunction (substance use or mental illness in the home), community violence (witnessing violence in their neighborhood or bullying), as well as exposure to pornography. See Figure 1 for the list of nineteen ACEs and the totals that answered yes or no. Of the 143 clients who responded to these questions, the distribution of ACE totals was as follows: 4.2% (*n* = 6) reported zero forms of trauma, 6.3% (*n* = 9) reported one, 4.2% (*n* = 6) reported two, 9.9% (*n* = 14) reported three, and 75.4% (*n* = 107) reported having experienced four or more forms of trauma before the age of 18.

Participants were given a Likert scale of 1-4 to rate the importance of focusing past traumas when in treatment for sex-offending behaviors (1 = not at all important and 4=extremely important). Of the respondents who answered this question (*n =* 151), one answered that it was “not at all important,” 26 (17.2%) responded that it was “somewhat important,” 58 (38.4%) stated that it was “very important,” and 66 (43.7%) said that it was “extremely important” for therapists to include a focus on past traumas when treating offending behaviors. The mean score was 3.25.

### Qualitative Results

During the qualitative analysis process, the research team found many overlapping themes across the two qualitative questions included in this study. Therefore, the themes that emerged across both questions are presented here. For each theme, we present the conceptual definition of the theme developed by the research team, along with quotes from the participants.

#### Strong Relationship Between Early Trauma and Subsequent Offending (n = 91)

This code was defined as the clients’ assertions that there is a strong connection between their trauma histories and their subsequent offending behaviors. When describing this connection, many reported that trauma histories and sexual offending were “100% linked” or “very integral” and that there was a “high correlation” between these experiences. Another said that their past traumatic experiences were “extremely important. In many cases, that is the original source of the problem….” Some went so far as to say that past traumas were the causative factor in their offense. For example, one client observed, “it can be the cause of the behaviors,” and another stated that, “for some offenders there appeared to be a very clear causal connection between their past trauma and offending behavior.” Although they were not asked directly about their own trauma histories and offenses, many discussed their own histories and offending. One client stated that, “if I wasn't abused in almost every facet of my young life I wouldn't have started offending. It was this abuse that drove me to offend in the end.” While there was widespread acknowledgement that not every traumatized person goes on to commit sexual offenses, respondents who voiced this theme often also observed this strong connection in men they met during their group treatment.

One common thesis suggested that offending was a repetition of their own past victimization. Several described how sex offenses repeated a cycle of abuse by “acting out what happened to them on others,” or “to repeat what they experienced in an attempt to work out their own abuse.” Another client was “convinced” that his offense “was a form of the type of situation replay seen in PTSD... A subconscious attempt to resolve the trauma(s) I experienced growing up. This does NOT excuse my offense, merely explains root drives.” Other clients hypothesized that their offense was a “learned behavior” that stemmed from early experiences. As one respondent wrote, “trauma is like anything else in life in that it teaches one how to act. That learned behavior becomes ingrained.” Another explained that “a person learns to believe abuse is ok if they've been abused.”

One participant shared his own story regarding the connection:

I developed a firm belief system by the time I was 14 that sex had no meaning at all. I didn’t believe any touch could become something different just because the touch happened on a “private part” instead of, say, your elbow. This of course contributed to a horrible and life altering decision because I didn’t see how I was hurting my victim.

Similarly, another stated: “I am a teen victim of rape by an older teen. I came up thinking of sex as nothing more than an activity. Rarely was it intimate for me.” The sentiments of many participants were captured by one participant’s statement that “Hurt people hurt other people.”

#### Acknowledging the Connection (n = 57)

Given the clients’ perception that there is a strong connection between trauma and subsequent offending, many participants wanted SOTX professionals to explicitly validate the linkages that clients believe exist. Many clients noted that their therapists rarely acknowledged any potential connections between trauma and offending. Some shared that when the topic of early abuse was raised by clients in treatment, therapists questioned their motives and characterized such conversation as a form of “manipulation” or as making “an excuse.” Ironically, participants viewed their attempts at understanding this connection as a way to help reduce their risk of recidivism. As one respondent stated, “trying to discuss one's trauma is to understand why the offense may have happened and to prevent another one, not to make excuses or try to garner pity.”

Participants reported that in treatment they wanted clinicians to listen and explore their histories by “digging deep into the past” to help them understand how and why past traumas can contribute to offending behaviors. One client noted that clinicians “need to know the scope and details of the trauma to better understand the situation of their offense.” Another participant indicated that it is important for professionals to explore the role of trauma because “…the link to (sexual) trauma might be the cause of the offending and using other paradigms (stressors in the year prior to offending, triggers, ‘hot buttons’) might miss the significant role that past trauma has played.”

Another aspect of this theme was the clients’ wish for their therapists to acknowledge that additional types of early developmental traumas (other than physical, sexual, or emotional abuse) have played a formative role in their lives, including poverty, being harassed or bullied, being LGBTQ+, mixed messages around sexuality, and early exposure to pornography. For example, one participant recalled that “the biggest thing I remember from group was that almost everyone had been exposed to pornography at a very young age. Some therapists would work with that, some would not.” Another client hypothesized that “Early perspectives of sexuality and sexual messaging can obscure an individual’s concept of appropriate sexual behavior.” For others, they described multiple traumas and life circumstances believed to have contributed to their sexual offenses. For example, one client shared:

Sex was a huge part of who I was, it was how I showed I "cared" - like the person who "cared" for me in my youth. Our home was broken and drug ridden, we were beyond poor. That person was my rock. This translated to even my adult (legal) relationships - sex was affection. In that way I became too close to my struggling teenage sister-in-law, and acted out in a way that fit with my rationalization at the time based on my past.

#### Specific Effects of Trauma (n = 48)

Many participants provided examples of how their past histories of trauma impacted them in very specific ways. This theme differed from the first, in that the participants described additional effects of trauma that had an indirect effect on their eventual offending. For example, they shared trauma impacts on their interpersonal capacities, such as a lack of adaptive decision-making or coping skills, as illustrated by one participant who noted that “Trauma can occur at any time in life and can influence bad behavior … if we don't have the coping mechanisms to prevent cognitive distortions.” This sentiment was shared by others who commented that “people with trauma lack life skills and coping skills for daily life.” Others noted a belief that their trauma led to struggles with rationality or/and judgement, as described by one client who stated that “for some, trauma, it can totally distort someone's thought process. It can condition an offender to think that what they are doing is normal.” These distortions also extended to their views about relationships, which one described as “warped” and in some cases permanently affected how individuals convicted of sexual offenses “see [them]selves and others and what sex is all about.”

Clients expressed a wish for clinicians to have specific expertise in understanding and treating the overall impacts and consequences of trauma. This sentiment was distinct from previous themes because they reflected the desire for providers to have clinical expertise to “know how to help people work through their past trauma” during SOTX. Respondents conveyed a need to move beyond validation; they wanted the expertise of their therapists to help them to understand the “why” behind their behavior. For example, one wanted knowledge about “the way that the young brain processes and reacts to trauma,” as they saw this knowledge to be “helpful for recovery.” Another wanted help understanding the social and relational impact of trauma, such as “trying to find a way to meet needs that were lacking as a child, no matter how irrational the thinking.”

#### Desire for Individualized Treatment (n = 34)

Group therapy is a standard modality in SOTX programs (McGrath et al., 2010), but many participants wished that interventions had been more individualized. They expressed that because not all clients have experienced the same “level of trauma,” nor do those with similar experiences have similar reactions, individualized treatment would allow for each client’s needs to be addressed more directly. For example, one client noted that the relationship between trauma and offending was unique and therefore was “totally dependent on circumstances and individuals involved.” Moreover, a few clients emphasized that they found discussing past trauma in group sessions to be “embarrassing” or otherwise “ineffective,” limiting their capacity or willingness to use group therapy to help them process their own traumas. They believed they would have benefited from a chance to speak with the therapist privately to explore their experiences.

In addition, they also argued that not all sexual offenses are the same and that “the term ‘sex offender’ is far too broad” for the wide spectrum of disparate sets of behaviors. As such, they questioned whether group or manualized treatments that were “one size fits all” could adequately address linkages between trauma and offending, or whether such treatments could effectively reduce recidivism. Those who noted in their open-ended responses that they had both individual and group sessions felt that individual sessions were “more beneficial,” and especially important for clients who had a trauma history.

#### Recognition of Humanity (n = 26)

Many respondents expressed a hope that SOTX professionals would see them as “human beings.” They used phrases such as wishes for therapists to be “caring and understanding,” “compassionate,” and “to listen to what we have to say.” These ideals contrasted with descriptions of being treated as if they were “deviant,” “monsters,” or “abnormal,” and they expressed a desire for therapists to find ways to help “lessen the shame” and treat them with “dignity and compassion” and “empathy.” In doing so, therapists could create a supportive, accepting, and “safe” environment to address past traumas and offenses simultaneously. Clients also wanted clinicians to convey hope for their treatment and recovery. As one client put it, “We are not broken people - just broken people in need of healing. Help us heal our past and we will be more likely to experience a healthy future.” In some cases, the participants noted that not only were these therapeutic components absent, but that their experiences in SOTX had caused them additional trauma, such as further damaging their trust in others. For example, one participant stated very simply that he wanted therapists “To listen to what we have to say.”

#### Additional Themes

Additional themes that emerged in the surveys also included “no justification” (*n =* 5), which was coded when participants indicated that past trauma must not be used as a justification for offense, or when participants perceived no connection at all between trauma and offending. Some clients (*n =* 12) expressed that they did not know or had no opinion on the topic. Others (*n =* 6) expressed that people who commit sex crimes do not always understand their own behavior.

## Discussion

The participants in this study endorsed the belief that there is a strong connection between their past traumas and their subsequent sexual offending. They also expressed the belief that traumas, whether from childhood or more recently, have influenced areas of functioning such as their interpersonal patterns and cognitive schemas. Given these connections, they expressed a wish that SOTX therapists would validate and acknowledge their trauma histories, while simultaneously helping clients to make meaning of past experiences as part of understanding their offense behavior. They opined that exploring and processing past trauma would aid them in developing new coping skills to avoid recidivism. Furthermore, given the unique experiences of individuals in SOTX, they suggested that programming should respond to the needs of each client in distinct and personalized ways. Finally, they expressed a strong belief that SOTX therapists should create a psychologically safe therapeutic environment where clients are treated humanely and holistically, with compassion and respect, and with a goal to instill hope for the future. The convergence of these themes is consistent with a need for SOTX providers to adopt a trauma-informed care (TIC) approach in their counseling services (Levenson, Willis, & Prescott, 2017). Our themes and recommendations are also concordant with those identified by Cartwright et al. (2022), who interviewed secure forensic service users; they endorsed the need for trauma-informed and reflective practices that consider attachment theories, relational approaches, and insight into developmental pathways to maladaptive schemas.

### Implications for Trauma-Informed Practice

Trauma should never be used to excuse or condone victimization of others. Trauma should be considered, however, as one piece of the puzzle when conceptualizing the factors that contribute to sex-offending behavior. There is no question that traumatic experiences, especially chronic childhood adversity, can alter developmental trajectories (Finkelhor & Kendall-Tackett, 1997; Levenson, Willis, & Prescott., 2017; Masten & Cicchetti, 2010; van der Kolk, 2005). In addition to a focus on risk and recidivism, there is value in discussing clients’ life experiences and their contributions to subsequent offending. Exploring trauma can lead to insights about the origins of unhealthy relational patterns, the maladaptive coping strategies often utilized by traumatized persons, and distorted thinking about self-identity, expectations of others, the world in general, and sexual matters more specifically (Bloom, 2013; Lambie & Reil, 2021; Najavits et al., 2009; Pettus-Davis et al., 2019).

Our participants noted that in many cases, their SOTX lacked individualized assessment and treatment planning, and they wished the therapeutic encounter had felt more safe, trustworthy, non-judgmental, and empowering; these qualities capture the cornerstones of TIC (Bloom, 2013; SAMHSA, 2014b). TIC is consistent with using an empirically-supported (Deming & Jennings, 2020) and risk-needs-responsivity approach (Bates-Maves & O’Sullivan, 2017; Deming & Jennings, 2020; Jung, 2017) that seeks to understand the unique factors which motivate and facilitate offending (Seto, 2019). Traumatic experiences contribute to dynamic risk through neuro-cognitive dysregulation, disrupted attachments, and role modelling of unhealthy interpersonal skills. We can promote protective factors and post-traumatic growth by responding to clients in ways that are empowering, collaborative, client-centered, and strengths-based (Levenson, Willis, & Prescott, 2017; Olver et al., 2020; Pettus-Davis et al., 2019; SAMHSA, 2014b; Ward & Fortune, 2016).

In keeping with the RNR approach, the goals of treatment should include tools for preventing reoffense as well as ensuring that clients have the tools they need to heal from their own past trauma and enhance their own well-being. RNR models begin by identifying dynamic risk factors, which connect to treatment needs such as skills deficits that often originated in traumagenic environments. For instance, dysregulation and maladaptive coping skills might represent responses to seemingly threatening conditions that re-activate hyperarousal, impulsivity, and cognitive problem-solving impairment – which, of course, increase risk for criminal behavior including sexually harmful acts. Responsivity provides flexibility in service delivery so that interventions can be individualized and relevant to each client’s risks, needs, and interpersonal style. These principles are consistent with a trauma-informed approach, which focuses on establishing a safe therapeutic alliance so that clients can develop the trust needed to begin the challenging and often painful work of self-exploration, personal growth, cognitive change, and internal regulation capacity. Integrating a trauma-informed framework into RNR principles would provide theoretical grounding through neuro-cognitive and attachment models of developmental psychopathology (Levenson, Willis, & Prescott, 2017; Masten & Cicchetti, 2010; van der Kolk, 2005). Adopting a holistic approach to SOTX would enhance interventions aimed at building, rehearsing, and enacting the skills clients need to prevent re-offense and live up to their full potential as human beings. Thus, we believe that a crucial element of reducing risk and improving resilience should include exploring trauma histories and making trauma-specific interventions available.

TIC is an universal approach to interventions that is guided by four primary principles (Substance Abuse and Mental Health Services Administration, 2014a) that can be applied to work with justice-involved clients: (1) *Recognize* the elevated prevalence of trauma and adversity in persons involved with the criminal justice system (Jäggi et al., 2016; Martin et al., 2015; Pettus-Davis et al., 2019); (2) *Realize* the ways that trauma contributes to criminogenic risk by considering its impacts on self-regulation, executive functioning, and interpersonal patterns (Ardino, 2012; Cheng et al., 2019; Holley et al., 2017; van der Kolk, 2006; Wojciechowski, 2020); (3) *Respond* by integrating trauma-informed and trauma-responsive interventions (Pettus-Davis et al., 2019); and (4) *Resist re-traumatizing* clients with unnecessarily confrontational and punitive treatments that fail to model empathy and respect (Blagden et al., 2016; Sachs & Miller, 2018; Stinson & Clark, 2017; Sturgess et al., 2016).

In addition, it is essential that all interventions be sensitive to and consider different identities in the delivery of care. For example, trauma-informed treatments should be gender-responsive; females have different needs and risks than males and placing them in groups with male offenders can re-activate trauma responses (Cortoni & Gannon, 2011; Covington & Bloom, 2007; Topitzes et al., 2011; Wijkman et al., 2010). As well, treatment should be culturally relevant and take into consideration the inter-generational and historical trauma of poverty, systemic racism, oppression, and discrimination (Jäggi et al., 2016; Sotero, 2006; St. Vil et al., 2019). Some authors have suggested that TIC does not go far enough to address structural inequality or adequately respond to the needs of individuals from marginalized populations (Ginwright, 2018). Ginwright advocates for Healing Centered Engagement (HCE), which highlights the collective harm by societal, political, and cultural institutions. While TIC tends to be centered around individual trauma, it can neglect root causes such as the political context of structural racism, the harm of living in violent communities, and the trauma of poverty. Thus, “culturally grounded” healing takes place collectively; it is strengths-focused – emphasizing how clients can become their best selves, and facilitates peer support to strengthen the roots of well-being. Clearly people who offend are responsible for those behaviors and the harm caused by them. Attributions of individual pathology offer limited pathways to change, however, without drawing attention to and acknowledging the contextual risk factors that foster traumatic experiences within some groups and communities.

SOTX therapists can create safe spaces with a trauma-responsive culture of healing so that clients feel able to be open and forthcoming. Skillful facilitation of group therapy (Sawyer & Jennings, 2016) can engage clients in helping each other make meaning of their experiences, thereby developing a sense of self-efficacy through the giving and receiving of mentoring and support (Nixon, 2020). In this way, clients can process trauma and improve interpersonal skills, paving the way for resilience and post-traumatic growth, and reducing dynamic risk factors for reoffending (Easton et al., 2013; Tedeschi et al., 2015). Trauma-informed practices can be infused into cognitive-behavioral programming with traditional SOTX goals of accepting responsibility, increasing empathy, correcting flawed thinking, building self-regulation skills, and devising plans to prevent re-offending (Bath & Seita, 2018; Steinkopf, 2021).

Implementing TIC is challenging, and transforming care requires clinicians to enable trauma-sensitive environments that promote healing, personal growth, and positive relationships (Bath & Seita, 2018; Steinkopf, 2021). Bath and Seita (2018) described the three pillars of TIC which begin with creating physical, emotional, relational, and cultural *safety*, which paves the way to reparative *connections* with the helping professional. The third pillar, *coping*, seeks to re-orient the client to more adaptive strategies to facilitate healthy thinking, behavior, social skills, and psycho-physiological management. However, these goals rely on a clinician’s ability to co-regulate (Steinkopf, 2021). In other words, clients with limited self-regulation capacity can easily contaminate the transactional dynamics within the therapeutic encounter, sparking dysregulated reactions by the clinician. Self-reflection and self-awareness are pre-requisites for therapists to actively model emotional grounding and avoid replication of alienating, hostile, or tumultuous relationship patterns. Through corrective experiences, therapists can help clients make sense of their experience and master self-help strategies for emotional, behavioral, and interpersonal management. To this end, SOTX training should offer a coherent and integrative neuroscientific and interpersonal theoretical model for understanding the impacts and manifestations of developmental trauma (Bath & Seita, 2018; Steinkopf, 2021). By solidifying the nexus between past experience and current attachment and relational problems, clinicians can better attune to client needs and successfully address longstanding patterns that may contribute to sexually abusive behavior.

Although many clinicians have gained awareness of TIC in recent years, the gap between their knowledge and their implementation of its principles was apparent in a recent study (Grady, Levenson, et al., 2021). Using the Trauma-Informed Principles Scale (C. M. Sullivan & Goodman, 2015) clinicians rated their adherence to TIC principles, and a similar scale was used for clients to rate their SOTX on the same items. On every item on the scale, clinicians rated themselves statistically significantly higher, meaning more aligned with TIC principles, as compared to how clients rated them. Not only did this study indicate that there is potentially a disconnect between what clinicians say they are doing and what may be happening in the field, some clients also reported that they were discouraged to talk about their own histories of trauma or experienced negative reactions from their SOTX when they did. The findings of this study indicate that SOTX may need additional training and resources to assist them in fully integrating TIC into their treatment programs.

### Implications for Policy and Research

Crime-prevention policies should strive to increase access to affordable mental health services so people who have experienced trauma can receive proper interventions before their risk levels escalate to a point of offending. It is in society’s interest to allocate resources for primary and secondary prevention strategies to strengthen at-risk families and communities, to improve trauma-informed responses to abused and neglected children, and to strengthen protective factors for marginalized groups who are most vulnerable to trauma (Larkin et al., 2014; Pettus-Davis & Epperson, 2015). Research funding is needed to explore the connection between trauma and criminality, as well as to study the effectiveness of integrated treatment approaches that incorporate trauma-specific interventions and TIC. Further, it is important to recognize how post-conviction sanctions may exacerbate dysregulation, interfering with successful re-integration and law-abiding lifestyle (D. A. Harris & Levenson, 2021).

### Limitations

The findings of this study should be considered along with its limitations. First, data obtained in this study were based on self-report, and there is no way to verify reported experiences or to control for variations in distant memory or recall. As well, people define trauma in different ways, and may differ in their perceptions of the experiences we asked about. Second, the survey did not ask the clients to identify the dates when they attended SOTX. Since dialog about TIC in SOTX began relatively recently, it is possible that SOTX programming more than 10 years ago was less likely to incorporate information about the role of trauma in the development of victimizing behaviors. Third, clients who receive SOTX may be mandated to do so after a sex crime conviction. As a result, they may have had negative feelings associated with treatment that was required as part of a criminal sentence, and their responses might have reflected those sentiments.

Fourth, we did not ask the clients to speak to their trauma specifically, but rather how in they viewed the connection between trauma and offending in general. As such it is possible that had we asked the question differently, we may have received different data for the analysis. Also, we did not stratify the sample based on race, gender, or any other identity and therefore were unable to identify if difference themes emerged between groups. Future research should seek to explore perceptions of different ethnic, racial, or gender groups regarding their treatment experiences that would inform clinicians working with this population.

Finally, it is possible that our study is unable to account for selection bias; perhaps those most likely to respond to the survey were those with higher rates of trauma in their past, or those more inclined to attribute their criminal behavior to past traumagenic factors. This sample was not demographically or geographically representative; minorities were under-represented in this study, as about 22% of registrants in the U.S. are black and about 12% are of a minority ethnicity (Ackerman et al., 2011). Furthermore, our recruitment methods probably excluded many without Internet access, despite our effort to provide access to a pen-and-paper mail-in survey option.

### Conclusion

People who commit sex offenses often have a history of traumatic life experiences. These must be considered by treatment providers in understanding risk and protective factors, and in formulating relevant treatment plans. A movement toward more trauma-informed SOTX contextualizes offending as a symptomatic constellation of underlying problems, rather than simply a behavior to be extinguished. Obviously prevention of future sex crimes is the most salient goal of treatment, and this objective might be more successfully achieved by using more trauma-informed RNR interventions.

## Data Availability

The data is available upon request from the lead author.
